# Association of polymorphisms with a family history of cancer and the presence of germline mutations in the BRCA1/BRCA2 genes

**DOI:** 10.1186/s13053-015-0042-1

**Published:** 2016-01-13

**Authors:** Gabriela C. Fernandes, Rodrigo A. D. Michelli, Cristovam Scapulatempo-Neto, Edenir I. Palmero

**Affiliations:** Molecular Oncology Research Center, Barretos Cancer Hospital, Barretos, Brazil; Post-Graduate Program in Oncology, Barretos Cancer Hospital, Barretos, Brazil; Oncogenetics Department, Barretos Cancer Hospital, Barretos, Brazil; Pathology Department, Barretos Cancer Hospital, Barretos, Brazil; Barretos School of Health Sciences, Dr. Paulo Prata – FACISB, Barretos, Brazil

## Abstract

**Introduction:**

Breast cancer (BC) is an important public health problem worldwide. In Brazil, breast cancer is the most frequently diagnosed tumor and the leading cause of cancer death in women. Hereditary cancer represents approximately 5 to 10 % of BC cases. Even outside the hereditary cancer context, the presence of polymorphisms acting as genetic modifiers may contribute to a better or worse prognosis. Not much is known about the hereditary BC epidemiology in Brazil or about the influence of polymorphisms on hereditary predisposition.

**Objective:**

This study examined the role of five different polymorphisms in four groups of women with BC: Group 1: women with a germline mutation in the *BRCA1/2* genes; Group 2: women with variants of uncertain significance in *BRCA1/2* and Group 3: women with no mutations in *BRCA1/2*.

**Patients and methods:**

The women included in groups 1, 2 and 3 were patients from the Department of Oncogenetics of the Barretos Cancer Hospital who had undergone genetic testing because of a clinical suspicion of hereditary predisposition syndrome. The constitutive DNA was analyzed for the presence of polymorphisms at rs2981582 (*FGFR2* gene); rs3803662 (*TNRC9)*; rs889312 (*MAP3K1)*; rs3817198 (*LSP1* gene); and rs13281615 (8q24). The analyses were performed using PCR amplification and bi-directional sequencing.

**Results:**

No differences were identified in the frequency of the polymorphisms that were analyzed among the three groups. However, some associations were identified, such as the occurrence of bilateral breast cancer and homozygosity for the G allele in rs13281615 as well as the correlation between the SNPs rs2981582 and rs13281615 and the number of cancer cases in the family. Regarding the G allele of rs13281615, we observed that the proportion of individuals who were homozygous for this allele increased with the number of generations affected by cancer, regardless of the group where the patients were included. Concerning the rs2981582 we could observe that individuals from group 1 and homozygous CC had fewer cancer (and also fewer breast cancer) cases. Regarding the hormone receptors, we observed an increased frequency in C homozygotes (rs3803662) among estrogen receptor-negative individuals from groups 1 and 3. For rs2981582 (FGFR2), we observed an increased frequency of the T allele in women who were positive for the estrogen and progesterone receptors regardless of the *BRCA1/2* mutational status (*p* = 0.020 and *p* = 0.014, respectively).

**Conclusion:**

The results presented here provide interesting data on the modifying effect of polymorphisms on a family history of cancer; this may be a variable to consider in the analysis of tumor diversity, and of the family history observed in families with hereditary breast cancer (even in those harboring the same type of genetic alteration).

**Electronic supplementary material:**

The online version of this article (doi:10.1186/s13053-015-0042-1) contains supplementary material, which is available to authorized users.

## Introduction

In Brazil, breast neoplasms are the main cause of death by cancer among women [1]. It is currently estimated that 5-10 % of the total number of breast cancer (BC) cases are hereditary. *BRCA1* and *BRCA2* are among the genes associated with hereditary breast cancer [2–4]. Women harboring a germline mutation in the *BRCA1* gene show a lifetime cumulative risk (LCR) between 44 and 68 % of developing breast cancer until 70 years of age. Moreover, the LCR for ovarian cancer in these patients is also significantly higher and may reach 60 % by 70 years of age [5]. The *BRCA2* gene, when altered, is responsible for approximately 30 to 40 % of all cases of hereditary breast cancer. The LCR for breast cancer in women harboring germline mutations in this gene is similar to the risk of carriers of germline mutations in *BRCA1* (44 to 68 % until 70 years of age), whereas the risk of ovarian cancer ranges from 11 to 40 % [5–8]. Families with mutations in *BRCA1/2* differ in terms of age at diagnosis, the number of family members affected, and tumor prognosis [9]. Currently, several reports in the literature point to the role of polymorphisms as genetic modifiers of the risk of cancer in families and as responsible factors for part of this diversity identified in the families with known mutations [10–12].

In 2007, a large study was conducted by Easton and collaborators [13] involving genome-wide association studies (GWAS) of 4,398 cases (women with breast cancer) and 4,316 controls, followed by a validation step involving 21,860 cases and 22,578 controls. This study identified single nucleotide polymorphisms (SNPs) in five loci that were associated with the risk of developing breast cancer: 1) rs2981582 (C > T) in gene *FGFR2,* which encodes a tyrosine kinase receptor that acts in the development of the mammary glands; 2) rs3803662 (C > T), localized in a region that includes *TNRC9*; 3) rs889312 (A > C), localized in a region that includes *MAP3K1* in addition to two putative genes, *MGC33648* and *MIER3*; 4) rs3817198 (T > C) in gene *LSP1;* and 5) rs13281615 (A > G), which resides in a region that does not include any known gene (8q24) but where variants associated with an increased risk of prostate and colorectal cancer have been identified. The authors correlated the presence of the above mentioned polymorphisms with histopathological characteristics such as positivity or negativity for the estrogen and progesterone hormone receptors, stage of tumor development, nodes, size, histology and stage at diagnosis. Among the five SNPs mentioned above, three (rs2981582, rs3803662 and rs889312) were significantly associated with an increased risk of breast cancer in estrogen receptor-positive individuals. Women who were TT homozygous in SNP rs3803662 and who had estrogen-negative tumors exhibited a risk of developing breast cancer that was 1.28-fold (95 % CI = 1.13–1.45) higher than women who were homozygous for the wild-type allele (present in 53 % of the controls). Besides, Hunter and colleagues [14] identified an association between the SNP rs2981582 and increased risk for breast cancer among postmenopausal white women in a study involving 1,145 cases of invasive breast cancer and 1,142 controls. This association was further validated in 1,176 cases and 2,072 controls from three additional studies.

Studies involving rs3817198 have found that the least frequent allele (C) leads to an increase of approximately 10 % in the risk of breast cancer in Caucasian women; however, the same allele has a protective effect among women of African ascent [15, 16].

Considering the factors described above and the possible modifying effect conferred by polymorphisms, the objective of this study was to evaluate the frequency of the polymorphisms rs2981582, rs3803662, rs889312, rs3817198*,* and rs13281615 in women with breast cancer, with or without mutations in the *BRCA1* and *BRCA*2 genes, and to correlate the frequency of the different polymorphisms with the family history of cancer in general and, more specifically with the history of breast and ovarian cancer and with the histopathological features of the tumors.

## Materials and Methods

### Cases

This project was approved by the Research Ethics Committee of the Barretos Cancer Hospital. All the participants in the study were treated at this institution and signed an informed consent form. The women from the oncogenetics department underwent genetic testing for mutations in the *BRCA1* and *BRCA2* genes and, based on the results, were allocated to three different groups: a) Group 1: 51 women with a personal and family history of breast cancer *with* a pathogenic germline mutation in the *BRCA1* and/or *BRCA2* genes; b) Group 2: 53 women with a personal and family history of breast cancer *with the presence* of a variant of unknown clinical significance (VUS) identified in the *BRCA1* and/or *BRCA2* genes; c) Group 3: 100 women with a personal and family history of breast cancer without a pathogenic mutation and/or mutation of unknown clinical significance identified in the *BRCA1* and/or *BRCA2* genes.

### Molecular Analysis

For the polymorphism analysis, DNA was extracted using the QIAamp Blood DNA Mini-Kit (Qiagen) according to the manufacturer's instructions. Genotyping was performed through PCR amplification using the Hot Start Taq enzyme (Qiagen). Following amplification, the products were purified with the ExoSap enzyme (USB products) and sequenced bidirectionally using the BigDye Terminator v3.1 Kit (Applied Biosystems, USA). Electrophoresis was run in the automated sequencer model 3500 (Applied Biosystems, USA).

For the analysis of mutations in the *BRCA1* and *BRCA2* genes and the subsequent separation of the participants into the three study groups, a multiplex PCR amplification of all coding exons of the *BRCA1* (NCBI; NM_007294.3) and *BRCA2* (NCBI; NM_000059.3) genes and their respective flanking intronic regions was performed, followed by bidirectional sequencing using two platforms (ABI 3500 XL sequencer) and a new generation sequencer (Ion Torrent PGM, Applied Biosystems). In addition, large rearrangements were investigated using the multiplex ligation-dependent probe amplification (MLPA) technique.

### Statistical Analysis

The program SPSS v.21.0 for Windows (Chicago, IL) was used for the statistical analysis. The categorical variables were described using absolute frequencies and relative percentage frequencies. The correlations were obtained using the Chi-square and Fisher’s exact tests. The level of significance adopted in all the tests was 5 %.

## Results

Sequencing of *BRCA1* and *BRCA2* of all coding regions was performed for the 204 patients. Fifty-one had a pathogenic mutation identified (30 with *BRCA1* mutations and 21 with a germline pathogenic mutation on *BRCA2* gene), 53 had a VUS identified on *BRCA1* and/or *BRCA2* genes and 100 women were tested negative for both genes. All 204 women had a personal history of breast cancer (mean age at diagnosis = 38.4 years). In addition all participants had a positive family history of cancer, suggestive of Hereditary Breast and Ovarian Cancer Predisposition Syndrome (for details regarding criteria used for genetic testing at Barretos Cancer Hospital see Palmero et al., “Oncogenetics service and the Brazilian public health system: the experience of a reference Cancer Hospital”, manuscript accepted for publication in 2015 at Genetics and Molecular Biology journal). The main histological tumor types were invasive ductal carcinomas (91.8 %) and lobular carcinomas (3.9 %). In addition the great majority of the breast tumors (of the probands) were grade II, T1/T2, N0 and M0. Details about the family history of cancer and mutated gene can be found in Additional file 1: Table S1.

The following polymorphisms were considered in this study: rs2981582 in gene *FGFR2* (allele frequency in the general population: T: 0.404, C: 0.596), rs3803662 in gene TNRC9 (allele frequency in the general population: T: 0.440, C: 0.560), rs889312 in gene MAP3K1 (allele frequency in the general population: C: 0.387, A: 0.613), rs3817198 in gene *LSP1* (allele frequency in the general population: T: 0.785, C: 0.215) and rs13281615 at 8q24 (allele frequency in the general population: A: 0.509, G: 0.491).

Table 1 presents the genotype frequency of the five polymorphisms that were evaluated and their distribution in the different study groups.

**Table 1:** Frequency of polymorphisms rs3803662 (*TNRC9)*, rs2981582 (*FGFR2)*, rs13281615, rs889312 (*MAP3K1)* and rs3817198 (*LSP1)* per group

Variable	Group 1	Group 2	Group 3	P value^a^
	N (%)	N (%)	N (%)	
rs3803662 (*TNRC9*)				**0.027**
TT	5 (9.8)	12 (22.6)	17 (17.3)	
CT	27 (52.9)	24 (45.3)	47 (48.0)	
CC	19 (37.3)	17 (32.1)	34 (34.7)	
rs2981582 (*FGFR2*)				**0.031**
TT	14 (27.5)	9 (16.7)	26 (26.3)	
CT	22 (43.1)	33 (61.1)	49 (49.5)	
CC	15 (29.4)	12 (22.2)	24 (24.2)	
rs13281615 (8q24)				**0.029**
AA	14 (27.5)	11 (20.4)	27 (27.0)	
AG	24 (47.1)	26 (48.1)	48 (48.0)	
GG	13 (25.5)	17 (31.5)	25 (25.0)	
rs889312 (*MAP3K1*)				**0.029**
CC	8 (15.7)	8 (14.8)	11 (11.0)	
CA	21 (41.2)	20 (37.0)	55 (55.0)	
AA	22 (43.1)	26 (48.1)	34 (34.0)	
rs3817198 (*LSP1*)				**0.023**
TT	26 (51.0)	28 (51.9)	44 (44.4)	
TC	22 (43.1)	24 (44.4)	43 (43.4)	
CC	3 (5.9)	2 (3.7)	12 (12.1)	

Group 1: women with germline mutations in the *BRCA1/BRCA2* genes; Group 2: women with VUS in the *BRCA1/BRCA2* genes; Group 3: women WT for the *BRCA1/BRCA2* genes; Group 4: control, sporadic group

Values in bold indicate statistical significance (*p* < 0.05)

^a^Chi-square

Although there were no marked differences in the frequency of the polymorphisms studied among the three groups, some findings are noteworthy: *i)* there was a higher frequency of TT homozygotes for rs3803662 in the groups comprising women without mutations in the *BRCA1/BRCA2* genes; *ii)* regarding rs889312, an increase was observed in the frequency of heterozygous individuals among those without mutations or VUS in *BRCA1/2*; and *iii*) the Group3 (WT) had a relatively increased frequency of CC homozygotes for the SNP rs3817198 compared to groups 1 and 2.

The analysis of the family history of cancer according to the different polymorphisms studied is detailed in Table 2.

**Table 2:** Correlations between the genotype frequencies of the polymorphisms and the family history of cancer (overall)

	Number of cancer cases		Presence of bilateral breast cancer		Presence of ovarian cancer		Number of generations with cancer				Number of breast cancer cases	
Polymorphism	≤3	>3	Yes	No	Yes	No	1	2	3	4	≤3	>3
rs3803662 – TT	13	21	4	30	4	30	8	17	8	1	25	9
N (%)	(20.0)	(15.3)	(20.0)	(16.5)	(14.3)	(17.2)	(22.2)	(17.9)	(12.5)	(16.7)	(17.6)	(15.3)
rs3803662 - TC	25	73	9	89	15	83	15	44	35	4	63	34
N (%)	(38.5)	(53.3)	(45.0)	(48.9)	(53.6)	(47.7)	(41.7)	(46.3)	(54.7)	(66.7)	(44.4)	(57.6)
rs3803662 - CC	27	43	7	63	9	61	13	34	21	1	54	16
N (%)	(41.5)	(31.4)	(35.0)	(34.6)	(32.1)	(35.1)	(36.1)	(35.8)	(32.8)	(16.7)	(38.0)	(27.1)
Total N (%)	65 (100.0)	137 (100.0)	20 (100.0)	182 (100)	28 (100)	245 (100.0)	36 (100)	95 (100)	64 (100)	6 (100)	142 (100)	59 (100)
**P value** ^a^	(*p* = 0.602)		(*p* = 0.849)		(*p* = 0.998)		(*p* = 0.890)				(*p* = 0.429)	
rs2981582 - TT	17	32	4	45	2	47	6	28	12	3	33	15
N (%)	(25.8)	(23.2)	(20.0)	(24.5)	(7.5)	(26.6)	(10.7)	(29,5)	(18,2)	(50,0)	(23,1)	(25,0)
rs2981582 - TC	28	76	13	91	18	86	16	44	42	1	70	34
N (%)	(42.4)	(55.1)	(65.0)	(49.5)	(66.6)	(48.6)	(44.4)	(46.3)	(63.6)	(16.7)	(59.0)	(56.7)
rs2981582 - CC	21	30	3	48	7	44	14	23	12	2	40	11
N (%)	(31.8)	(21.7)	(15.0)	(26.1)	(25.9)	(24.9)	(38.9)	(24,2)	(18.2)	(33.3)	(28.0)	(18.3)
Total N (%)	120	156	20	184	27	177	36	95	66	6	143	60
	(100.0)	(100)	(100.0)	(100)	(100)	(100.0)	(100)	(100)	(100)	(100)	(100)	(100)
**P value** ^a^	(*p* = 0.475)		(*p* = 0.688)		(*p* = 0.163)		(*p* = 0.172)				(*p* = 0.283)	
rs13281615 - AA	21	31	3	49	11	41	14	22	16	0	37	15
N (%)	(31.8)	(22.3)	(15.0)	(26.5)	(39.3)	(23.2)	(38.9)	(23.2)	(23.9)	(0.0)	(25.7)	(25.0)
rs13281615 - AG	37	61	5	93	12	86	16	51	27	3	70	27
N (%)	(56.1)	(43.9)	(25.0)	(50.3)	(42.8)	(48.6)	(44.4)	(53.7)	(40.3)	(50.0)	(48.6)	(45.0)
rs13281615 -GG	8	47	12	43	5	50	6	22	24	3	37	18
N (%)	(12.1)	(33.8)	(60.0)	(23.2)	(17.9)	(28.2)	(16.7)	(23,2)	(35.8)	(50.0)	(25.7)	(30.0)
Total N (%)	66	139	20	185	28	177	36	95	67	6	144	60
	(100)	(100)	(100.0)	(100)	(100)	(100)	(100)	(100)	(100)	(100)	(100)	(100)
**P value** ^a^	**0.004**		**0.005**		0.072		**0.007**				0.654	
N (%)	(16.7)	(11.5)	(25.0)	(11.9)	(7.1)	(14.1)	(11.1)	(14.7)	(13.4)	(0.0)	(14.6)	(10.0)
rs889312 - CA	28	68	12	84	12	84	18	36	36	5	66	30
N (%)	(42.4)	(48.9)	(60.0)	(45.4)	(42.9)	(47.5)	(50.0)	(37.9)	(53.7)	(83.3)	(45.8)	(50.0)
rs889312 - AA	27	55	3	79	14	68	14	45	22	1	57	24
N (%)	(40.9)	(39.6)	(15.0)	(42.7)	(50.0)	(38.4)	(38.9)	(47.4)	(32.8)	(16.7)	(39.6)	(40.0)
Total N (%)	_66 (100)_	139	20	185	28	177	36	95	67	6	144	60
		(100)	(100)	(100)	(100)	(100)	(100)	(100)	(100)	(100)	(100)	(100)
**P value** ^a^	0.707		**0.011**		0.179		0.373				0.632	
rs3817198 - TT	31	67	6	92	18	80	21	41	33	2	70	28
N (%)	(47.0)	(48.6)	(30.0)	(50.0)	(66.7)	(45.2)	(58.3)	(43.2)	(50.0)	(33.3)	(49.0)	(46.7)
rs3817198 - TC	25	64	10	79	6	83	13	48	24	4	61	28
N (%)	(37.9)	(46.4)	(50.0)	(42.9)	(22.2)	(46.9)	(36.1)	(50.5)	(36.4)	(66.7)	(42.7)	(46.7)
rs3817198 - CC	10	7	4	13	3	14	2	6	9	0	12	4
N (%)	(15.2)	(5.1)	(20.0)	(7.1)	(11.1)	(7.9)	(5.6)	(6.3)	(13.6)	(0.0)	(8.4)	(6.6)
Total N (%)	66	138	20	184	27	177	36	95	66	6	143	60
	(100)	(100)	(100)	(100)	(100)	(100)	(100)	(100)	(100)	(100)	(100)	(100)
**P value** ^a^	0.223		**0.029**		0.166		0.275				0.954	

Values in bold indicate statistical significance (*p* < 0.05)

^a^Chi-square

When comparing the genotypes with the family history of cancer, some potential associations were identified, including the occurrence of bilateral breast cancer and homozygosity for the G allele in rs13281615 and “C” in rs889312 in which 85 % of women with bilateral breast cancer had at least one C allele (or G allele in the case of rs13281615). Likewise, 85 % of the women with bilateral breast cancer had at least one T allele in rs2981582. Furthermore, regarding allele G in rs13281615, we observed that the proportion of individuals who were homozygous for this allele increased with the number of generations affected by cancer. Moreover, we observed that in the case of rs3803662 and rs2981582, heterozygous TC individuals presented a higher number of cancer cases (in general) and a higher number of breast cancer cases.

Detailed data on the frequency of the different genotypes versus the family history of cancer with the women subgrouped according to their *BRCA1* and *BRCA2* gene mutation status are found in Additional file 1: Table S2 to S6.

In addition, the frequency of the different genotypes was compared to the family history by separately considering the women with a mutation in *BRCA1* to those with mutations in *BRCA2*. Given the limited sample size of patients with a deleterious mutation identified in these genes, the group stratification per mutated gene limited the statistical analyses that were conducted. However, some trends could be observed, for example, all of the women with mutations in the *BRCA2* gene with bilateral breast cancer were homozygous for the G allele in rs132281615, and among those with mutations in *BRCA1* and with bilateral breast cancer, 86 % had at least one G allele. Moreover, in regard to rs3817198, all of the women with mutations in *BRCA1* and with ovarian cancer had at least one T allele (with 88.9 % being TT homozygotes); however, the same pattern was not observed for the women with mutations in *BRCA2* and a personal history of ovarian cancer (*p* = 0.006). To reach a conclusion on the association of these polymorphisms with the *BRCA1/2* mutational status and with a family history of cancer, more patients with these characteristics must be genotyped.

The data correlating the genotypes of the five polymorphisms that were analyzed with the status of the hormone receptors (estrogen, progesterone and HER2) are shown in Table 3.

**Table 3:** Correlation between the genotype frequencies of the polymorphisms and the hormone receptors (overall)

	Estrogen		Progesterone		Her-2		
Polymorphisms	Negative	Positive	Negative	Positive	Negative	Positive	Inconclusive
rs3803662 - TT N (%)	13 (16.5)	20 (18.0)	16 (18.0)	17 (16.5)	23 (16.9)	7 (14.9)	3 (100.0)
rs3803662 - TC N (%)	33 (41.8)	56 (50.5)	51 (41.8)	38 (42,7)	66 (48.5)	21 (44.7)	0 (0.0)
rs3803662 – CC N (%)	33 (41.8)	35 (31.5)	46 (37.7)	35 (39.3)	47 (34.6)	19 (40.4)	0 (0.0)
Total	79 (100.0)	111 (100.0)	122 (100.0)	89 (100.0)	136 (100.0)	47 (100.0)	3 (100.0)
**P value** ^**a**^	0.257		0.504		0.469		
rs2981582 – TT N (%)	15 (219.2)	31 (27.2)	16 (18.0)	30 (28.6)	33 (23.9)	21 (25.5)	1 (33.4)
rs2981582 – TC	38 (48.7)	60 (52.6)	47 (52.8)	52 (49.5)	65 (47.1)	28 (59.6)	2 (66.6)
N (%)							
rs2981582 – CC	25 (32.1)	23 (20.2)	26 (29.2)	23 (21.9)	40 (29.0)	7 (14.9)	0 (0.0)
^N (%)^							
Total	78 (100.0)	114 (100.0)	89 (100.0)	105 (100.0)	138 (100.0)	47 (100.0)	3 (100.0)
**P value** ^**a**^	0.054		0.076		0.120		
rs13281615 - AA N (%)	20 (25.3)	30 (26.3)	26 (28.9)	25 (23.8)	32 (23.2)	14 (29.2)	2 (66.6)
rs13281615 - AG N (%)	35 (44.3)	56 (49.1)	40 (44.4)	51 (48.6)	68 (49.3)	20 (41.7)	1 (33.4)
rs13281615 - GG N (%)	24 (30.4)	28 (24.6)	24 (26.7)	29 (27.6)	38 (27.5)	14 (29.2)	0 (0.0)
Total	79 (100.0)	114 (100.0)	90 (100.0)	105 (100.0)	138 (100.0)	48 (100.0)	3 (100.0)
P value^a^	0.523		0.566		0.291		
rs889312 - CC N (%)	12 (15.2)	15 (13.2)	10 (11,1)	17 (16.2)	21 (15.2)	5 (10.4)	0 (0.0)
rs889312 - CA N (%)	35 (44.3)	54 (47.4)	45 (50.0)	45 (42.9)	61 (44.2)	25 (52.1)	1 (33.3)
rs889312 - AA N (%)	32 (40.5)	45 (39.5)	35 (38.9)	43 (41.0)	56 (40.6)	18 (37.5)	2 (66.7)
Total	79 (100.0)	114 (100.0)	90 (100.0)	105 (100.0)	138 (100.0)	48 (100.0)	3 (100.0)
**P value** ^**a**^	0.921		0.760		0.554		
rs3817198 - TT N (%)	42 (53.8)	51 (44.7)	49 (55.1)	45 (42,9)	68 (49.3)	21 (44.7)	0 (0.0)
rs3817198 - TC N (%)	31 (39,7)	54 (47.4)	34 (38.2)	52 (49,5)	59 (42.8)	23 (48.9)	3 (100)
rs3817198 - CC N (%)	5 (6.4)	9 (7.9)	6 (6.7)	8 (7.6)	11 (8.0)	3 (6.4)	0 (0.0)
Total	78 (100.0)	114 (100.0)	89 (100.0)	105 (100.0)	138 (100.0)	47 (100.0)	3 (100.0)
**P value** ^**a**^	0.248		0.145		0.449		

Values in bold indicate statistical significance (*p* < 0.05)

^a^Chi-square

When the different polymorphisms were analyzed with the hormone receptors, we observed an increased frequency of heterozygotes for rs3803662 in individuals with estrogen receptor (ER)-positive tumors, in individuals with progesterone receptor (PR)- positive tumors and in individuals who were negative for the HER2 receptor. Regarding rs2981582, individuals with at least one T allele more frequently exhibited positivity for the three receptors (ER, PR and HER2). We also observed an increased frequency of the WT allele among ER-negative individuals. For the SNPs rs13281615, rs889312 and rs3817198, no association was found between their frequencies and the hormone receptors considered.

When the analysis of the polymorphisms with the hormone receptors was conducted separately for the groups studied (Additional file 1: Table S6 to S10), we noted some associations. Regarding the SNP rs2981582 in gene *FGFR2*, we observed an association between group 2 (with VUS in the *BRCA1/2* genes) with the ER in which the presence of TT homozygotes was not observed, and TC heterozygosity was present in 73.7 % of the ER- negative cases (Additional file 1: Table S8). Additionally, when this analysis was performed for the SNP rs13281615, we observed an association with HER-2 positivity in group 3 (WT for *BRCA1/2*), with 50 % of individuals being AG heterozygotes vs. 25 % and 28.6 % of those from groups one and two respectively (Additional file 1: Table S9). For the SNP rs889312 in gene *MAP3K1*, an association with HER-2-negative tumors was observed in group 2, and 46.0 % of the negative cases were homozygous for the A allele (Additional file 1: Table S10). The SNP rs3817198 in gene *LSP1* showed an association with the ER in group 3 (WT), in which 50.0 % of the ER-positive cases were heterozygous (TC) (Additional file 1: Table S11). Finally, in the case of the SNP rs3803662 in gene *TNRC9,* the increased frequency of allele C homozygosity was more evident in groups 1 and 3, in which most cases of ER-negative tumors have the CC genotype (Additional file 1: Table S7).

An analysis of genotypes versus hormone receptors was conducted by separately considering the individuals with *BRCA1* mutations and those with identified mutations in *BRCA2*, but no association was observed in this analysis (data not shown).

The association between the genotypes in the five SNPs considered and the age at diagnosis was evaluated. Although no significant association was identified, these data are shown in Additional file 1: Table S12.

## Discussion

Genetic polymorphisms are being widely evaluated as modifiers of the genetic risk of disease by several research groups. A study conducted by Garcia-Closas and collaborators [17] evaluated the frequency of five polymorphisms that were previously reported to be associated with an increased risk of breast cancer [13] and investigated whether the association between these polymorphisms and the risk of breast cancer was somehow influenced by clinically relevant tumoral features in 23,039 cases of invasive breast cancer and in 26,273 controls from 20 different studies. The authors found that common genetic variants (polymorphisms) influenced the pathological tumor subtype, which provided additional evidence of the biologically distinct behavior of ER-positive versus ER- negative tumors.

In this study, we compared the genotype distribution of the five polymorphisms that were previously reported in the literature to be associated with the risk of breast cancer [13, 17, 18] in three groups of women: women at-risk for hereditary breast cancer, with germline mutations in the *BRCA1* and *BRCA2* genes, women with VUS identified in one of those genes, and women WT for both genes. The genotype data from the five polymorphisms were then correlated with the family history of cancer and the histopathological features of the tumors, always considering a possible influence of the mutational status of *BRCA1/2*. A similar work was performed by Antoniou and collaborators in 2008,[19] where they genotyped *FGFR2* (rs2981582), *TNRC9* (rs3803662), and *MAP3K1* (rs889312) SNPs in a sample of 10,358 mutation carriers from 23 studies.

When we compared the genotypes of the polymorphisms analyzed with the pathological characteristics of the tumors, we noted that, in the case of the SNP rs3803662 in gene *TNRC9*, there was an increased frequency of CC homozygotes among ER-negative individuals (as previously reported by Easton and collaborators [13] in 2007), and although the difference was not statistically significant, it was possible to observe that this difference was stronger among women in groups 1 (with mutations) and 3 (WT). Regarding the SNP rs2981582, which was localized in gene *FGFR2*, we observed an increased frequency of the T allele in women who were positive for the estrogen and progesterone receptors (*p* = 0.020 and *p* = 0.014, respectively); this increase in frequency among individuals positive for the hormone receptors (ER and PR) did not depend on the group to which the women belonged. The same association with the rs2981582 and positivity for estrogen and progesterone receptors was described by Garcia-Closas and collaborators in an analysis of the association among breast cancer risk, *FGFR2* rs2981582 and clinical and pathological characteristics and they found that the effect of rs2981582 was stronger in ER/PR-positive patients [17]. Additionally, Liang and colleagues [20] in a work involving 1049 breast cancer Chinese patients and 1073 cancer-free controls found the same association. The authors stated that this association possibly can be due to the interaction of *FGFR2* variants with high levels of endogenous sex hormones (FGFR2 is upregulated in ERα-positive breast tumors) [21], especially estrogens, which may increase breast cancer risk.

There are several studies evaluating the effects of polymorphisms on the risk of cancer and their association with tumor features. According to the study published by Tapper and collaborators [22] in 2008, some polymorphisms capable of altering the risk of breast cancer are being discovered. The study conducted by the above mentioned authors had the objective of evaluating how those variants may influence the prognosis and risk of developing breast cancer. For this purpose, 1,001 women with non-familial, invasive breast cancer that was diagnosed at early age were analyzed compared to a group of women with hereditary breast cancer; the presence and frequency of 206 SNPs in 30 candidate genes were evaluated in the two groups. An association with an increased risk of developing breast cancer was found in the SNPs localized in the *CASP8, TNRC9* and *ESR1* genes. The authors also reported an association between survival and eight SNPs in six genes (*MAP3K1, DAPK1, LSP1, MMP7, TOX3* and *ESR1*) and another SNP in a gene-free region in 8q24. For the SNPs in genes *MMP7, TOX3* and *MAP3K1*, the survival effects did not depend on the known main clinical prognosis factors. The effect of the SNP in ESR1 on survival was more significant when only ER-positive tumors were evaluated. When the cases were stratified according to tumor features, it was observed that the SNPs in *FGFR2* and *TOX3* were associated with the disease. Finally, the study showed that several SNPs were associated with survival. In some cases, this association may occur because of an effect on the tumor characteristics that has an impact on prognosis; in other cases, the effect appears to be independent of these prognostic factors [22].

Results published by Fanale and collaborators [11] in 2012 demonstrated the association of some SNPs with breast cancer. The genes in which the polymorphisms described reside in are *TNRC9, FGFR2, MAP3K1, H19* and *LSP1*. The most strongly associated SNP is localized in gene *FGFR2,* which was overexpressed in 5-10 % of the cases of breast cancer. The SNP in gene *TNRC9* showed a stronger association with breast cancer and appeared to be correlated with the presence of bone metastases and positivity for the estrogen receptor. The SNP rs889312 in gene *MAP3K1* showed sensitivity only in individuals harboring mutations in *BRCA2*, and it was not associated with an increased risk in carriers of *BRCA1* mutations. Several SNPs in *LSP1* and *H19* were most likely associated with the risk of developing cancer. Therefore, the study concluded that the identification of risk-modifying polymorphisms may lead to a better understanding of the biological mechanism of breast cancer, thus improving the prevention, detection and early treatment of the disease.

The results of the analyses of the presence and frequency of the five polymorphisms considered here were compared to the family history of cancer. Several associations were identified in that comparison, either with the number of cancer cases in the family (associated with the SNPs rs2981582 and rs13281615), with the presence of bilateral breast cancer (SNP rs13281615) or with the number of generations affected by cancer (SNP rs13281615). Moreover, regarding allele G of rs13281615, we observed that the proportion of individuals who were homozygous for this allele increased as the number of generations affected by cancer increased. Many of the findings described here corroborate data in the literature, such as the association found between SNP rs13281615 and a positive family history of cancer, which was a correlation previously reported by Gorodnova and collaborators in 2010 [23]. Besides, Gong et al. [24] published a meta-analysis that included 14 studies involving 44,283 cases and 55,756 controls found that the GG and G-allele genotypes of rs13281615 at 8q24 are significantly associated with increased risk for breast cancer

The effect of polymorphisms on the family history of cancer was also reported by Huijts and collaborators [25], who showed an association between rs2981582 in gene *FGFR2* and the average number of first- and second-degree relatives with breast and/or ovarian cancers (*P* = 0.05). The authors also found an association with the age at diagnosis, in which individuals who were heterozygous or homozygous for the least frequent allele for rs3803662 had a higher probability of having the cancer diagnosed at an age younger than 60 years.

An association with age at diagnosis was not observed in this study for any of the five polymorphisms considered. However, we should reinforce that these findings should be regarded with caution, given the relatively small size of our study, which is the main limitation of our study. For that reason the obtained data and the possible associations identified with the tumor pathological features and with the family history of cancer must be validated using a larger sample size. Nonetheless, if these associations are confirmed, we will have additional data to understand the epidemiology of hereditary and familial cancer in our population. In addition, the results contributed to the continued research efforts in the search for common variants for breast cancer that could help to explain the high variability observed among family history of families with hereditary susceptibility to breast cancer.

Thus, although no significant association between the presence of the 5 analyzed polymorphisms and the occurrence of pathogenic mutation in *BRCA1*/*BRCA2* has been identified, the influence of polymorphisms acting as genetic modifiers of cancer was observed, especially for the SNP rs13281615 whose presence the G allele was associated with the occurrence of bilateral breast cancer, as well as with an increased number of cancer cases in the family and with an increasing number of generations affected by cancer and, this association was, in general, independent of the mutational status of the *BRCA1* or *BRCA2* genes.

## Conclusion

Some potential associations were found between the five polymorphisms analyzed in this study previously reported in the literature as being associated and an increase in the risk of breast cancer, with an emphasis placed on the effect of these polymorphisms on the family history of cancer and on hormone receptor positivity/negativity. These findings must be interpreted with caution because of the limited sample size in this study. The data must be validated with a larger number of cases and, if confirmed, the clinical relevance of the associations identified and their applicability in medical practice must be considered carefully. However, the results presented here provide interesting information on the modifying effect of polymorphisms on the family history of cancer and may be a variable to consider in the analysis of tumor diversity, and of the family history observed in families with hereditary breast cancer (even in those harboring the same type of genetic alteration).

## Additional file

Additional file 1:
**Supplementary Tables.** (DOCX 122 kb)
