# Supplementary material for: Association of polymorphisms with a family history of cancer and the presence of germline mutations in the BRCA1/BRCA2 genes
Source: Hered Cancer Clin Pract. 2016 Jan 13;14:2. doi: 10.1186/s13053-015-0042-1 (PMC4712611; doi:10.1186/s13053-015-0042-1)
Supplement: Additional file 1: — Supplementary Tables. (DOCX 122 kb) [file 13053_2015_42_MOESM1_ESM.docx]

**Additional file 1**

**Supplementary Tables**

**Table S1.** Mutation status and family history of cancer.

| **Mutated Gene** | **Exon** | **Mutation Type** | **Presence of BBC*** | **Presence of OC*** | **Presence of MBC*** | **Number of BCC**** |
| --- | --- | --- | --- | --- | --- | --- |
| BRCA1 | 11 | Frameshift | no | no | no | >3 cases |
| BRCA1 | 16 | Frameshift | yes | no | no | ≤3 cases |
| BRCA1 | 20 | Frameshift | yes | no | no | >3 cases |
| BRCA1 | 5 to 7 | Deletion | no | yes | no | ≤3 cases |
| BRCA1 | 19 | Frameshift | no | yes | no | ≤3 cases |
| BRCA1 | 5 | Missense | yes | yes | no | >3 cases |
| BRCA1 | 8 | Frameshift | no | no | no | >3 cases |
| BRCA1 | 20 | Frameshift | yes | yes | no | ≤3 cases |
| BRCA1 | 20 | Frameshift | no | no | no | >3 cases |
| BRCA1 | 11 | Nonsense | no | no | no | >3 cases |
| BRCA1 | 5 | Missense | no | no | no | ≤3 cases |
| BRCA1 | 12 | Frameshift | no | no | no | ≤3 cases |
| BRCA1 | 13 | Splicing | no | yes | no | >3 cases |
| BRCA1 | 20 | Frameshift | yes | no | no | ≤3 cases |
| BRCA1 | 11 | Frameshift | no | yes | no | ≤3 cases |
| BRCA1 | 17 | Frameshift | no | no | no | >3 cases |
| BRCA1 | 20 | Frameshift | no | no | no | >3 cases |
| BRCA1 | 11 | Nonsense | no | no | no | ≤3 cases |
| BRCA1 | 20 | Frameshift | no | no | no | ≤3 cases |
| BRCA1 | 20 | Frameshift | no | no | no | >3 cases |
| BRCA1 | 23 | Frameshift | no | yes | no | >3 cases |
| BRCA1 | 20 | Nonsense | no | yes | no | >3 cases |
| BRCA1 | 20 | Nonsense | no | yes | no | >3 cases |
| BRCA1 | 20 | Frameshift | no | no | no | >3 cases |
| BRCA1 | 11 | Frameshift | no | no | no | ≤3 cases |
| BRCA1 | 11 | Frameshift | no | no | no | >3 cases |
| BRCA1 | 20 | Frameshift | yes | no | no | >3 cases |
| BRCA1 | 20 | Frameshift | yes | no | no | >3 cases |
| BRCA1 | 11 | Nonsense | no | no | no | ≤3 cases |
| BRCA1 | 23 | Nonsense | no | no | no | >3 cases |
| BRCA2 | 20 | Splicing | no | no | no | ≤3 cases |
| BRCA2 | 8 | Frameshift | no | yes | no | ≤3 cases |
| BRCA2 | 11 | Frameshift | no | no | no | >3 cases |
| BRCA2 | 11 | Frameshift | no | no | no | >3 cases |
| BRCA2 | 11 | Frameshift | no | yes | no | ≤3 cases |
| BRCA2 | 11 | Frameshift | no | no | no | ≤3 cases |
| BRCA2 | 11 | Frameshift | no | no | no | ≤3 cases |
| BRCA2 | 11 | Nonsense | no | no | no | >3 casos |
| BRCA2 | 19 | Missense | yes | no | no | ≤3 cases |
| BRCA2 | 21 | Frameshift | no | yes | no | >3 cases |
| BRCA2 | 18 | Frameshift | yes | no | no | >3 cases |
| BRCA2 | 10 | Missense | no | no | no | >3 cases |
| BRCA2 | 14 | Nonsense | no | no | no | >3 cases |
| BRCA2 | 25 | Frameshift | no | no | no | >3 cases |
| BRCA2 | 25 | Nonsense | no | yes | no | ≤3 cases |
| BRCA2 | 2 | Missense | no | no | no | >3 cases |
| BRCA2 | 10 | Frameshift | no | no | no | ≤3 cases |
| BRCA2 | 11 | Missense | no | no | no | ≤3 cases |
| BRCA2 | 2 | Missense | no | no | no | >3 cases |
| BRCA2 | 19 | Missense | no | no | no | ≤3 cases |
| BRCA2 | 11 | Frameshift | yes | no | no | ≤3 cases |

*Presence in the proband or in the family; **Total number in the family; BBC= bilateral breast cancer; OC= ovarian cancer; MBC= male bilateral breast cancer; BCC= breast cancer cases.

**Table S2** Correlation between the frequency of polymorphism rs3803662 in gene *TNRC9* and the family history of cancer (per group).

| **Family history** | **TT**  **N (%)** | **TC**  **N (%)** | **CC**  **N (%)** | **Total**  **N (%)** | **P value*** |
| --- | --- | --- | --- | --- | --- |
| **Number of cancer cases** |  |  |  |  |  |
| Group 1 |  |  |  |  | 0.235 |
| ≤3 | 2 (25.0) | 2 (25.0) | 4 (50.0) | 8 (100.0) |  |
| >3 | 3 (7.0) | 25 (58.1) | 15 (34.9) | 43 (100.0) |  |
| Group 2 |  |  |  |  | 0.149 |
| ≤3 | 6 (33.3) | 5 (27.7) | 7 (38.9) | 18 (100.0) |  |
| >3 | 6 (17.1) | 19 (54.3) | 10 (28.6) | 35 (100.0) |  |
| Group 3 |  |  |  |  | 0.055 |
| ≤3 | 5 (12.8) | 18 (46.2) | 16 (41.0) | 39 (100.0) |  |
| >3 | 12 (20.3) | 29 (49.2) | 18 (30.5) | 59 (100.0) |  |
| **Presence of bilateral breast cancer** |  |  |  |  |  |
| Group 1 |  |  |  |  | 0.198 |
| Yes | 1 (10.0) | 6 (60.0) | 3 (30.0) | 10 (100.0) |  |
| No | 4 (9.8) | 21 (51.2) | 16 (39.0) | 41 (100.0) |  |
| Group 2 |  |  |  |  | 0.234 |
| Yes | 2 (40.0) | 1 (20.0) | 2 (40.0) | 5 (100.0) |  |
| No | 10 (20.8) | 23 (48.0) | 15 (31.2) | 48 (100.0) |  |
| Group 3 |  |  |  |  | 0.253 |
| Yes | 1 (20.0) | 2 (40.0) | 2 (40.0) | 5 (100.0) |  |
| No | 16 (17.2) | 45 (48.4) | 32 (34.4) | 93 (100.0) |  |
| **Presence of ovarian cancer** |  |  |  |  |  |
| Group 1 |  |  |  |  | 0.098 |
| Yes | 0 (0.0) | 7 (53.8) | 6 (46.2) | 13 (100.0) |  |
| No | 5 (13.2) | 20 (52.6) | 13 (34.2) | 38 (100.0) |  |
| Group 2 |  |  |  |  | 0.175 |
| Yes | 1 (25.0) | 3 (75.5) | 0 (0.0) | 4 (100.0) |  |
| No | 11 (22.5) | 21 (42.9) | 17 (34.6) | 49 (100.0) |  |
| Group 3 |  |  |  |  | 0.122 |
| Yes | 3 (27.3) | 5 (45.4) | 3 (27.3) | 11 (100.0) |  |
| No | 14 (16.1) | 42 (48.3) | 31 (35.6) | 87 (100.0) |  |
| **Number of generations with cancer** |  |  |  |  |  |
| Group 1 |  |  |  |  | 0.086 |
| 1 | 1 (11.1) | 3 (33.3) | 5 (55.6) | 9 (100.0) |  |
| 2 | 4 (20.0) | 8 (40.0) | 8 (40.0) | 20 (100.0) |  |
| 3 | 0 (0.0) | 13 (68.4) | 6 (31.6) | 19 (100.0) |  |
| 4 | 0 (0.0) | 3 (100.0) | 0 (0.0) | 3 (100.0) |  |
| Group 2 |  |  |  |  | **0.039** |
| 1 | 5 (45.5) | 4 (36.4) | 2 (18.1) | 11 (100.0) |  |
| 2 | 4 (16.0) | 12 (48.0) | 9 (36.0) | 25 (100.0) |  |
| 3 | 3 (17.7) | 8 (47.0) | 6 (35.3) | 17 (100.0) |  |
| 4 | 0 (0.0) | 0 (0.0) | 0 (0.0) | 0 (0.0) |  |

**Table S2 (Cont.):** Correlation between the frequency of polymorphism rs3803662 in gene *TNRC9* and the family history of cancer (per group).

| **Family history** | **TT**  **N (%)** | **TC**  **N (%)** | **CC**  **N (%)** | **Total**  **N (%)** | **P value*** |
| --- | --- | --- | --- | --- | --- |
| **Number of generations with cancer** |  |  |  |  |  |
| Group 3 |  |  |  |  | 0.066 |
| 1 | 2 (12.5) | 8 (50.0) | 6 (37.5) | 16 (100.0) |  |
| 2 | 9 (18.0) | 24 (48.0) | 17 (34.0) | 50 (100.0) |  |
| 3 | 5 (17.9) | 14 (50.0) | 9 (32.1) | 28 (100.0) |  |
| 4 | 1 (33.3) | 1 (33.3) | 1 (33.4) | 3 (100.0) |  |
| **Number of breast cancer cases** |  |  |  |  |  |
| Group 1 |  |  |  |  | 0.089 |
| ≤3 | 3 (13.0) | 8 (34.8) | 12 (52.2) | 23 (100.0) |  |
| >3 | 2 (7.1) | 19 (67.9) | 7 (25.0) | 28 (100.0) |  |
| Group 2 |  |  |  |  | 0.167 |
| ≤3 | 11 (25.6) | 18 (41.9) | 14 (32.5) | 43 (100.0) |  |
| >3 | 1 (10.0) | 6 (60.0) | 3 (30.0) | 10 (100.0) |  |
| Group 3 |  |  |  |  | 0.061 |
| ≤3 | 11 (14.5) | 37 (48.7) | 28 (36.8) | 76 (100.0) |  |
| >3 | 6 (28.6) | 9 (42.8) | 6 (28.6) | 21 (100.0) |  |
| **Age at diagnosis** |  |  |  |  |  |
| Group 1 |  |  |  |  | **0.041** |
| ≤30 years | 0 (0.0) | 4 (50.0) | 4 (50.0) | 8 (100.0) |  |
| >30 and ≤50 years | 4 (11.8) | 16 (47.0) | 14 (41.2) | 34 (100.0) |  |
| >50 years | 1 (11.1) | 7 (77.8) | 1 (11.1) | 9 (100.0) |  |
| Group 2 |  |  |  |  | 0.083 |
| ≤30 years | 4 (16.7) | 14 (58.3) | 6 (25.0) | 24 (100.0) |  |
| >30 and ≤50 years | 7 (29.2) | 10 (41.6) | 7 (29.2) | 24 (100.0) |  |
| >50 years | 1 (20.0) | 0 (0.0) | 4 (80.0) | 5 (100.0) |  |
| Group 3 |  |  |  |  | **0.004** |
| ≤30 years | 3 (10.0) | 12 (40.0) | 15 (50.0) | 30 (100.0) |  |
| >30 and ≤50 years | 9 (16.6) | 28 (51.9) | 17 (31.5) | 54 (100.0) |  |
| >50 years | 4 (30.8) | 7 (53.9) | 2 (15.3) | 13 (100.0) |  |

*Chi-square/ Fisher’s exact

**It was not possible to conduct a comparative analysis in group 4 for the following variables: presence of bilateral breast cancer, presence of ovarian cancer and age at diagnosis.

**Note:** Values in bold indicate statistical significance (p<0.05).

**Table S3:** Correlation between the frequency of polymorphism rs2981582 in gene *FGFR2* and the family history of cancer (per group).

| **Family history** | **TT**  **N (%)** | **TC**  **N (%)** | **CC**  **N (%)** | **Total**  **N (%)** | **P value*** |
| --- | --- | --- | --- | --- | --- |
| **Number of cancer cases** |  |  |  |  |  |
| Group 1 |  |  |  |  | 0.074 |
| ≤3 | 2 (25.0) | 1 (12.5) | 5 (62.5) | 8 (100.0) |  |
| >3 | 12 (27.9) | 21 (48.8) | 10 (23.3) | 43 (100.0) |  |
| Group 2 |  |  |  |  | 0.164 |
| ≤3 | 4 (22.2) | 10 (55.6) | 4 (22.2) | 18 (100.0) |  |
| >3 | 5 (13.9) | 23 (63.9) | 8 (22.2) | 36 (100.0) |  |
| Group 3 |  |  |  |  | 0.100 |
| ≤3 | 11 (27.5) | 17 (42.5) | 12 (30.0) | 40 (100.0) |  |
| >3 | 15 (25.4) | 32 (54.2) | 12 (20.4) | 59 (100.0) |  |
| **Presence of bilateral breast cancer** |  |  |  |  |  |
| Group 1 |  |  |  |  | 0.111 |
| Yes | 3 (30.0) | 6 (60.0) | 1 (10.0) | 10 (100.0) |  |
| No | 11 (26.8) | 16 (39.0) | 14 (34.2) | 41 (100.0) |  |
| Group 2 |  |  |  |  | 0.257 |
| Yes | 0 (0.0) | 4 (80.0) | 1 (20.0) | 5 (100.0) |  |
| No | 9 (18.3) | 29 (59.2) | 11 (22.5) | 49 (100.0) |  |
| Group 3 |  |  |  |  | 0.249 |
| Yes | 1 (20.0) | 3 (60.0) | 1 (20.0) | 5 (100.0) |  |
| No | 25 (26.6) | 46 (48.9) | 23 (24.5) | 94 (100.0) |  |
| **Presence of ovarian cancer** |  |  |  |  |  |
| Group 1 |  |  |  |  | 0.129 |
| Yes | 2 (15.4) | 7 (53.9) | 4 (30.7) | 13 (100.0) |  |
| No | 12 (31.6) | 15 (39.5) | 11 (28.9) | 38 (100.0) |  |
| Group 2 |  |  |  |  | 0.269 |
| Yes | 0 (0.0) | 3 (75.0) | 1 (25.0) | 4 (100.0) |  |
| No | 9 (18.0) | 30 (60.0) | 11 (22.0) | 50 (100.0) |  |
| Group 3 |  |  |  |  | 0.111 |
| Yes | 0 (0.0) | 8 (80.0) | 2 (20.0) | 10 (100.0) |  |
| No | 26 (29.2) | 41 (46.1) | 22 (24.7) | 89 (100.0) |  |
| **Number of generations with cancer** |  |  |  |  |  |
| Group 1 |  |  |  |  | 0.077 |
| 1 | 2 (22.2) | 3 (33.3) | 4 (44.5) | 9 (100.0) |  |
| 2 | 8 (40.0) | 6 (30.0) | 6 (30.0) | 20 (100.0) |  |
| 3 | 2 (10.5) | 13 (68.4) | 4 (21.1) | 19 (100.0) |  |
| 4 | 2 (66.7) | 0 (0.0) | 1 (33.3) | 3 (100.0) |  |
| Group 2 |  |  |  |  | 0.109 |
| 1 | 1 (9.1) | 6 (54.5) | 4 (36.4) | 11 (100.0) |  |
| 2 | 6 (24.0) | 15 (60.0) | 4 (16.0) | 25 (100.0) |  |
| 3 | 2 (11.1) | 12 (66.6) | 4 (22.3) | 18 (100.0) |  |
| 4 | 0 (0.0) | 0 (0.0) | 0 (0.0) | 0 (0.0) |  |

**Table S3 (Cont.):** Correlation between the frequency of polymorphism rs2981582 in gene *FGFR2* and the family history of cancer (per group).

| **Family history** | **TT**  **N (%)** | **TC**  **N (%)** | **CC**  **N (%)** | **Total**  **N (%)** | **P value*** |
| --- | --- | --- | --- | --- | --- |
| **Number of generations with cancer** |  |  |  |  |  |
| Group 3 |  |  |  |  | **0.035** |
| 1 | 3 (18.7) | 7 (43.8) | 6 (37.5) | 16 (100.0) |  |
| 2 | 14 (8.4) | 23 (13.7) | 13 (77.9) | 50 (100.0) |  |
| 3 | 8 (27.6) | 17 (58.6) | 4 (13.8) | 29 (100.0) |  |
| 4 | 1 (33.3) | 1 (33.3) | 1 (33.4) | 3 (100.0) |  |
| **Number of breast cancer cases** |  |  |  |  |  |
| Group 1 |  |  |  |  | 0.063 |
| ≤3 | 6 (26.1) | 7 (30.4) | 10 (43.5) | 23 (100.0) |  |
| >3 | 8 (28.6) | 15 (53.6) | 5 (17.8) | 28 (100.0) |  |
| Group 2 |  |  |  |  | 0.162 |
| ≤3 | 9 (20.5) | 25 (56.8) | 10 (22.7) | 44 (100.0) |  |
| >3 | 0 (0.0) | 8 (66.6) | 4 (33.4) | 10 (100.0) |  |
| Group 3 |  |  |  |  | 0.087 |
| ≤3 | 18 (23.7) | 38 (50.0) | 20 (26.3) | 76 (100.0) |  |
| >3 | 7 (31.8) | 11 (50.0) | 4 (18.2) | 22 (100.0) |  |
| **Age at diagnosis** |  |  |  |  |  |
| Group 1 |  |  |  |  | 0.125 |
| ≤30 years | 2 (25.0) | 4 (50.0) | 2 (25.0) | 8 (100.0) |  |
| >30 and ≤50 years | 10 (29.4) | 13 (38.2) | 11 (32.4) | 34 (100.0) |  |
| >50 years | 2 (22.2) | 5 (55.6) | 2 (22.2) | 9 (100.0) |  |
| Group 2 |  |  |  |  | 0.080 |
| ≤30 years | 6 (25.0) | 12 (50.0) | 6 (25.0) | 24 (100.0) |  |
| >30 and ≤50 years | 3 (12.0) | 18 (72.0) | 4 (16.0) | 25 (100.0) |  |
| >50 years | 0 (0.0) | 3 (60.0) | 2 (40.0) | 5 (100.0) |  |
| Group 3 |  |  |  |  | 0.085 |
| ≤30 years | 10 (33.3) | 12 (40.0) | 8 (26.7) | 30 (100.0) |  |
| >30 and ≤50 years | 13 (23.2) | 31 (55.4) | 12 (21.4) | 56 (100.0) |  |
| >50 years | 3 (25.0) | 6 (50.0) | 3 (25.0) | 12 (100.0) |  |

*Chi-square/ Fisher’s exact

**It was not possible to conduct a comparative analysis in Group 4 for the following variables: presence of bilateral breast cancer, presence of ovarian cancer and number of breast cancer cases.

**Note:** Values in bold indicate statistical significance (p<0.05).

**Table S4:** Correlation between the frequency of polymorphism rs13281615 and the family history of cancer (per group).

| **Family history** | **AA**  **N (%)** | **AG**  **N (%)** | **GG**  **N (%)** | **Total**  **N (%)** | **P value*** |
| --- | --- | --- | --- | --- | --- |
| **Number of cancer cases** |  |  |  |  |  |
| Group 1 |  |  |  |  | 0.133 |
| ≤3 | 2 (25.0) | 6 (75.0) | 0 (0.0) | 8 (100.0) |  |
| >3 | 12 (27.9) | 18 (41.8) | 13 (33.3) | 43 (100.0) |  |
| Group 2 |  |  |  |  | **0.003** |
| ≤3 | 7 (41.2) | 8 (47.0) | 2 (11.8) | 18 (100.0) |  |
| >3 | 4 (11.1) | 17 (47.2) | 15 (41.7) | 36 (100.0) |  |
| Group 3 |  |  |  |  | **0.039** |
| ≤3 | 12 (30.0) | 22 (55.0) | 6 (15.0) | 40 (100.0) |  |
| >3 | 15 (25.0) | 26 (43.3) | 19 (31.7) | 60 (100.0) |  |
| **Presence of bilateral breast cancer** |  |  |  |  |  |
| Group 1 |  |  |  |  | **0.008** |
| Yes | 1 (10.0) | 3 (30.0) | 6 (60.0) | 41 (100.0) |  |
| No | 13 (31.7) | 21 (51.2) | 7 (17.1) | 10 (100.0) |  |
| Group 2 |  |  |  |  | **0.020** |
| Yes | 0 (0.0) | 1 (20.0) | 4 (80.0) | 5 (100.0) |  |
| No | 11 (22.5) | 25 (51.0) | 13 (26.5) | 49 (100.0) |  |
| Group 3 |  |  |  |  | 0.245 |
| Yes | 2 (40.0) | 1 (20.0) | 2 (40.0) | 95 (100.0) |  |
| No | 25 (26.3) | 47 (49.5) | 23 (24.2) | 5 (100.0) |  |
| **Presence of ovarian cancer** |  |  |  |  |  |
| Group 1 |  |  |  |  | 0.086 |
| Yes | 5 (38.5) | 6 (46.1) | 2 (15.4) | 13 (100.0) |  |
| No | 9 (23.7) | 18 (47.3) | 11 (29.0) | 38 (100.0) |  |
| Group 2 |  |  |  |  | 0.167 |
| Yes | 1 (25.0) | 3 (75.0) | 0 (0.0) | 4 (100.0) |  |
| No | 10 (20.0) | 23 (46.0) | 17 (34.0) | 50 (100.0) |  |
| Group 3 |  |  |  |  | 0.130 |
| Yes | 5 (45.4) | 3 (27.3) | 3 (27.3) | 11 (100.0) |  |
| No | 22 (24.7) | 45 (50.6) | 22 (24.7) | 89 (100.0) |  |
| **Number of generations with cancer** |  |  |  |  |  |
| Group 1 |  |  |  |  | 0.090 |
| 1 | 2 (22.2) | 4 (44.5) | 3 (33.3) | 9 (100.0) |  |
| 2 | 3 (15.0) | 14 (70.0) | 3 (15.0) | 20 (100.0)) |  |
| 3 | 9 (47.4) | 5 (26.3) | 5 (26.3) | 19 (100.0) |  |
| 4 | 0 (0.0) | 1 (33.3) | 2 (66.7) | 3 (100.0) |  |
| Group 2 |  |  |  |  | **0.003** |
| 1 | 6 (54.6) | 4 (36.3) | 1 (9.1) | 11 (100.0) |  |
| 2 | 3 (12.0) | 14 (56.0) | 8 (32.0) | 25 (100.0) |  |
| 3 | 2 (1.2) | 8 (44.4) | 8 (44.4) | 18 (100.0) |  |
| 4 | 0 (0.0) | 0 (0.0) | 0 (0.0) | 0 (0.0) |  |

**Table S4 (Cont.):** Correlation between the frequency of polymorphism rs13281615 and the family history of cancer (per group).

| **Family history** | **AA**  **N (%)** | **AG**  **N (%)** | **GG**  **N (%)** | **Total**  **N (%)** | **P value*** |
| --- | --- | --- | --- | --- | --- |
| **Number of generations with cancer** |  |  |  |  |  |
| Group 3 |  |  |  |  | **0.005** |
| 1 | 6 (37.5) | 8 (50.0) | 2 (12.5) | 16 (100.0) |  |
| 2 | 16 (32.0) | 23 (46.0) | 11 (22.0) | 50 (100.0) |  |
| 3 | 5 (16.6) | 14 (46.7) | 11 (36.7) | 30 (100.0) |  |
| 4 | 0 (0.0) | 2 (66.7) | 1 (33.3) | 3 (100.0) |  |
| **Number of breast cancer cases** |  |  |  |  |  |
| Group 1 |  |  |  |  | 0.148 |
| ≤3 | 5 (21.7) | 14 (60.9) | 4 (17.4) | 23 (100.0) |  |
| >3 | 9 (32.1) | 10 (35.8) | 9 (32.1) | 28 (100.0) |  |
| Group 2 |  |  |  |  | 0.130 |
| ≤3 | 10 (22.7) | 21 (47.7) | 13 (29.6) | 44 (100.0) |  |
| >3 | 1 (10.0) | 5 (50.0) | 4 (40.0) | 10 (100.0) |  |
| Group 3 |  |  |  |  | 0.130 |
| ≤3 | 22 (28.6) | 35 (45.4) | 20 (26.0) | 77 (100.0) |  |
| >3 | 5 (22.7) | 12 (54.6) | 5 (22.7) | 22 (100.0) |  |
| **Age at diagnosis** |  |  |  |  |  |
| Group 1 |  |  |  |  | **0.034** |
| ≤30 years | 4 (50.0) | 4 (50.0) | 0 (0.0) | 8 (100.0) |  |
| >30 and ≤50 years | 7 (20.5) | 18 (53.0) | 9 (26.5) | 34 (100.0) |  |
| >50 years | 3 (33.3) | 2 (22.2) | 4 (44.5) | 9 (100.0) |  |
| Group 2 |  |  |  |  | 0.097 |
| ≤30 years | 8 (33.3) | 8 (33.3) | 8 (33.4) | 24 (100.0) |  |
| >30 and ≤50 years | 2 (8.0) | 15 (60.0) | 8 (32.0) | 25 (100.0) |  |
| >50 years | 1 (20.0) | 3 (60.0) | 1 (20.0) | 5 (100.0) |  |
| Group 3 |  |  |  |  | 0.065 |
| ≤30 years | 11 (36.6) | 13 (43.4) | 6 (20.0) | 30 (100.0) |  |
| >30 and ≤50 years | 13 (23.2) | 27 (48.2) | 16 (28.6) | 56 (100.0) |  |
| >50 years | 3 (23.1) | 8 (61.5) | 2 (15.4) | 13 (100.0) |  |

*Chi-square/ Fisher’s exact

**It was not possible to conduct a comparative analysis in group 4 for the following variables: presence of bilateral breast cancer, presence of ovarian cancer and number of breast cancer cases.

**Note:** Values in bold indicate statistical significance (p<0.05).

**Table S5:** Correlation between the frequency of polymorphism rs889312 in gene *MAP3K1* and the family history of cancer (per group).

| **Family history** | **CC**  **N (%)** | **CA**  **N (%)** | **AA**  **N (%)** | **Total**  **N (%)** | **P value*** |
| --- | --- | --- | --- | --- | --- |
| **Number of cancer cases** |  |  |  |  |  |
| Group 1 |  |  |  |  | 0.205 |
| ≤3 | 2 (25.0) | 2 (25.0) | 4 (50.0) | 8 (100.0) |  |
| >3 | 6 (14.0) | 19 (44.1) | 18 (41.9) | 43 (100.0) |  |
| Group 2 |  |  |  |  | 0.077 |
| ≤3 | 4 (22.2) | 7 (38.9) | 7 (38.9) | 18 (100.0) |  |
| >3 | 4 (11.1) | 13 (36.1) | 19 (52.8) | 36 (100.0) |  |
| Group 3 |  |  |  |  | 0.109 |
| ≤3 | 5 (12.5) | 19 (47.5) | 16 (40.0) | 40 (100.0) |  |
| >3 | 6 (10.0) | 36 (60.0) | 18 (30.0) | 60 (100.0) |  |
| **Presence of bilateral breast cancer** |  |  |  |  |  |
| Group 1 |  |  |  |  | 0.079 |
| Yes | 2 (20.0) | 6 (60.0) | 2 (20.0) | 10 (100.0) |  |
| No | 6 (14.6) | 15 (36.6) | 20 (48.8) | 41 (100.0) |  |
| Group 2 |  |  |  |  | **0.019** |
| Yes | 2 (40.0) | 3 (60.0) | 0 (0.0) | 5 (100.0) |  |
| No | 6 (12.2) | 17 (34.7) | 26 (53.1) | 49 (100.0) |  |
| Group 3 |  |  |  |  | 0.199 |
| Yes | 1 (20.0) | 3 (60.0) | 1 (20.0) | 5 (100.0) |  |
| No | 10 (10.5) | 52 (54.7) | 33 (34.8) | 95 (100.0) |  |
| **Presence of ovarian cancer** |  |  |  |  |  |
| Group 1 |  |  |  |  | 0.147 |
| Yes | 1 (7.6) | 6 (46.2) | 6 (46.2) | 13 (100.0) |  |
| No | 7 (18.4) | 15 (39.5) | 16 (42.1) | 38 (100.0) |  |
| Group 2 |  |  |  |  | 0.164 |
| Yes | 0 (0.0) | 1 (25.0) | 3 (75.0) | 4 (100.0) |  |
| No | 8 (16.0) | 19 (38.0) | 23 (46.0) | 50 (100.0) |  |
| Group 3 |  |  |  |  | 0.156 |
| Yes | 1 (9.0) | 5 (45.5) | 5 (45.5) | 11 (100.0) |  |
| No | 10 (11.2) | 50 (56.2) | 29 (32.6) | 89 (100.0) |  |
| **Number of generations with cancer** |  |  |  |  |  |
| Group 1 |  |  |  |  | 0.088 |
| 1 | 0 (0.0) | 6 (66.7) | 3 (33.3) | 9 (100.0) |  |
| 2 | 5 (25.0) | 4 (20.0) | 11 (55.0) | 20 (100.0) |  |
| 3 | 3 (15.8) | 9 (47.3) | 7 (36.9) | 19 (100.0) |  |
| 4 | 0 (0.0) | 2 (66.4) | 1 (33.3) | 3 (100.0) |  |
| Group 2 |  |  |  |  | 0.081 |
| 1 | 3 (27.3) | 3 (27.3) | 5 (45.4) | 11 (100.0) |  |
| 2 | 3 (12.0) | 10 (40.0) | 12 (48.0) | 25 (100.0) |  |
| 3 | 2 (11.1) | 7 (38.9) | 9 (50.0) | 18 (100.0) |  |
| 4 | 0 (0.0) | 0 (0.0) | 0 (0.0) | 0 (0.0) |  |

**Table S5 (Cont.):** Correlation between the frequency of polymorphism rs889312 in gene *MAP3K1* and the family history of cancer (per group).

| **Family history** | **CC**  **N (%)** | **CA**  **N (%)** | **AA**  **N (%)** | **Total**  **N (%)** | **P value*** |
| --- | --- | --- | --- | --- | --- |
| **Number of generations with cancer** |  |  |  |  |  |
| Group 3 |  |  |  |  | **0.023** |
| 1 | 1 (6.3) | 9 (56.2) | 6 (37.5) | 16 (100.0) |  |
| 2 | 6 (12.0) | 22 (44.0) | 22 (44.0) | 50 (100.0) |  |
| 3 | 4 (13.3) | 20 (66.7) | 6 (20.0) | 30 (100.0) |  |
| 4 | 0 (0.0) | 3 (100.0) | 0 (0.0) | 3 (100.0) |  |
| **Number of breast cancer cases** |  |  |  |  |  |
| Group 1 |  |  |  |  | 0.103 |
| ≤3 | 5 (21.8) | 9 (39.1) | 9 (39.1) | 23 (100.0) |  |
| >3 | 3 (10.7) | 12 (42.8) | 13 (46.5) | 28 (100.0) |  |
| Group 2 |  |  |  |  | 0.146 |
| ≤3 | 7 (15.9) | 17 (38.6) | 20 (45.5) | 44 (100.0) |  |
| >3 | 1 (10.0) | 3 (30.0) | 6 (60.0) | 10 (100.0) |  |
| Group 3 |  |  |  |  | 0.116 |
| ≤3 | 9 (11.7) | 40 (51.9) | 28 (36.4) | 77 (100.0) |  |
| >3 | 2 (9.1) | 15 (68.2) | 5 (22.7) | 22 (100.0) |  |
| **Age at diagnosis** |  |  |  |  |  |
| Group 1 |  |  |  |  | 0.121 |
| ≤30 years | 1 (12.5) | 3 (37.5) | 4 (50.0) | 8 (100.0) |  |
| >30 and ≤50 years | 6 (17.6) | 13 (38.2) | 15 (44.2) | 34 (100.0) |  |
| >50 years | 1 (11.1) | 5 (55.5) | 3 (33.4) | 9 (100.0) |  |
| Group 2 |  |  |  |  | **0.030** |
| ≤30 years | 3 (12.5) | 7 (29.1) | 14 (58.4) | 24 (100.0) |  |
| >30 and ≤50 years | 3 (12.0) | 11 (44.0) | 11 (44.0) | 25 (100.0) |  |
| >50 years | 2 (40.0) | 2 (40.0) | 1 (20.0) | 5 (100.0) |  |
| Group 3 |  |  |  |  | 0.085 |
| ≤30 years | 5 (16.7) | 13 (43.3) | 12 (40.0) | 30 (100.0) |  |
| >30 and ≤50 years | 4 (7.1) | 34 (60.8) | 18 (32.1) | 56 (100.0) |  |
| >50 years | 2 (15.4) | 8 (61.5) | 3 (23.1) | 13 (100.0) |  |

*Chi-square/ Fisher’s exact

**It was not possible to conduct a comparative analysis in group 4 for the following variables: presence of bilateral breast cancer, presence of ovarian cancer and number of breast cancer cases.

**Note:** Values in bold indicate statistical significance (p<0.05).

**Table S6:** Correlation between the frequency of polymorphism rs3817198 in gene *LSP1* and the family history of cancer (per group).

| **Family history** | **TT**  **N (%)** | **TC**  **N (%)** | **CC**  **N (%)** | **Total**  **N (%)** | **P value*** |
| --- | --- | --- | --- | --- | --- |
| **Number of cancer cases** |  |  |  |  |  |
| Group 1 |  |  |  |  | 0.067 |
| ≤3 | 2 (25.0) | 5 (62.5) | 1 (12.5) | 8 (100.0) |  |
| >3 | 24 (55.9) | 17 (39.5) | 2 (4.6) | 43 (100.0) |  |
| Group 2 |  |  |  |  | 0.196 |
| ≤3 | 10 (55.5) | 7 (38.9) | 1 (5.5) | 18 (100.0) |  |
| >3 | 18 (50.0) | 17 (47.2) | 1 (2.8) | 36 (100.0) |  |
| Group 3 |  |  |  |  | 0.100 |
| ≤3 | 19 (47.5) | 13 (32.5) | 8 (20.0) | 40 (100.0) |  |
| >3 | 25 (42.4) | 30 (50.8) | 4 (6.8) | 59 (100.0) |  |
| **Presence of bilateral breast cancer** |  |  |  |  |  |
| Group 1 |  |  |  |  | 0.081 |
| Yes | 3 (30.0) | 6 (60.0) | 1 (10.0) | 41 (100.0) |  |
| No | 23 (56.1) | 16 (39.0) | 2 (4.9) | 10 (100.0) |  |
| Group 2 |  |  |  |  | 0.292 |
| Yes | 2 (40.0) | 3 (60.0) | 0 (0.0) | 5 (100.0) |  |
| No | 26 (53.0) | 21 (42.9) | 2 (4.1) | 49 (100.0) |  |
| Group 3 |  |  |  |  | **0.017** |
| Yes | 1 (20.0) | 1 (20.0) | 3 (60.0) | 5 (100.0) |  |
| No | 43 (45.7) | 42 (44.7) | 9 (9.6) | 94 (100.0) |  |
| **Presence of ovarian cancer** |  |  |  |  |  |
| Group 1 |  |  |  |  | **0.018** |
| Yes | 11 (84.6) | 1 (7.7) | 1 (7.7) | 13 (100.0) |  |
| No | 15 (39.4) | 21 (55.3) | 2 (5.3) | 38 (100.0) |  |
| Group 2 |  |  |  |  | 0.249 |
| Yes | 3 (75.0) | 1 (25.0) | 0 (0.0) | 4 (100.0) |  |
| No | 25 (50.0) | 23 (46.0) | 2 (4.0) | 50 (100.0) |  |
| Group 3 |  |  |  |  | 0.156 |
| Yes | 4 (40.0) | 4 (40.0) | 2 (20.0) | 10 (100.0) |  |
| No | 40 (45.0) | 39 (43.8) | 10 (11.2) | 89 (100.0) |  |
| **Number of generations with cancer** |  |  |  |  |  |
| Group 1 |  |  |  |  | 0.103 |
| 1 | 6 (66.7) | 2 (22.2) | 1 (11.1) | 9 (100.0) |  |
| 2 | 9 (45.0) | 10 (50.0) | 1 (5.0) | 20 (100.0) |  |
| 3 | 10 (52.6) | 8 (42.1) | 1 (5.3) | 19 (100.0) |  |
| 4 | 1 (33.3) | 2 (66.7) | 0 (0.0) | 3 (100.0) |  |
| Group 2 |  |  |  |  | **0.042** |
| 1 | 4 (36.4) | 6 (54.5) | 1 (9.1) | 11 (100.0) |  |
| 2 | 12 (48.0) | 13 (52.0) | 0 (0.0) | 25 (100.0) |  |
| 3 | 12 (66.6) | 5 (27.8) | 1 (5.6) | 18 (100.0) |  |
| 4 | 0 (0.0) | 0 (0.0) | 0 (0.0) | 0 (0.0) |  |

**Table S6 (Cont.):** Correlation between the frequency of polymorphism rs3817198 in gene *LSP1* and the family history of cancer (per group).

| **Family history** | **TT**  **N (%)** | **TC**  **N (%)** | **CC**  **N (%)** | **Total**  **N (%)** | **P value*** |
| --- | --- | --- | --- | --- | --- |
| **Number of generations with cancer** |  |  |  |  |  |
| Group 3 |  |  |  |  | **0.007** |
| 1 | 11 (68.8) | 5 (31.2) | 0 (0.0) | 16 (100.0) |  |
| 2 | 20 (40.0) | 25 (50.0) | 5 (10.0) | 50 (100.0) |  |
| 3 | 11 (37.9) | 11 (37.9) | 7 (24.2) | 29 (100.0) |  |
| 4 | 1 (33.3) | 2 (66.7) | 0 (0.0) | 3 (100.0) |  |
| **Number of breast cancer cases** |  |  |  |  |  |
| Group 1 |  |  |  |  | 0.175 |
| ≤3 | 13 (56.5) | 8 (34.8) | 2 (8.7) | 23 (100.0) |  |
| >3 | 13 (46.4) | 14 (50.0) | 1 (3.6) | 28 (100.0) |  |
| Group 2 |  |  |  |  | 0.195 |
| ≤3 | 21 (47.7) | 22 (50.0) | 1 (2.3) | 44 (100.0) |  |
| >3 | 7 (70.0) | 2 (20.0) | 1 (10.0) | 10 (100.0) |  |
| Group 3 |  |  |  |  | 0.124 |
| ≤3 | 36 (47.3) | 31 (40.8) | 9 (11.9) | 76 (100.0) |  |
| >3 | 8 (36.4) | 12 (54.5) | 2 (9.1) | 22 (100.0) |  |
| **Age at diagnosis** |  |  |  |  |  |
| Group 1 |  |  |  |  | 0.100 |
| ≤30 years | 6 (75.0) | 1 (12.5) | 1 (12.5) | 8 (100.0) |  |
| >30 and ≤50 years | 16 (47.0) | 17 (50.0) | 1 (3.0) | 34 (100.0) |  |
| >50 years | 4 (44.4) | 4 (44.4) | 1 (11.2) | 9 (100.0) |  |
| Group 2 |  |  |  |  | 0.109 |
| ≤30 years | 10 (41.7) | 13 (54.1) | 1 (4.2) | 24 (100.0) |  |
| >30 and ≤50 years | 15 (60.0) | 10 (40.0) | 0 (0.0) | 25 (100.0) |  |
| >50 years | 3 (60.0) | 1 (20.0) | 1 (20.0) | 5 (100.0) |  |
| Group 3 |  |  |  |  | 0.085 |
| ≤30 years | 17 (56.7) | 11 (36.7) | 2 (6.6) | 30 (100.0) |  |
| >30 and ≤50 years | 20 (35.7) | 27 (48.2) | 9 (16.1) | 56 (100.0) |  |
| >50 years | 7 (58.3) | 5 (41.7) | 0 (0.0) | 12 (100.0) |  |

**Table S7:** Correlation between the genotype frequency of polymorphism rs3803662 in gene *TNRC9* and the hormone receptors (per group).

| **Hormone Receptors** | **TT**  **N (%)** | **TC**  **N (%)** | **CC**  **N (%)** | **Total**  **N (%)** | **P value*** |
| --- | --- | --- | --- | --- | --- |
| **Estrogen** |  |  |  |  |  |
| Group 1 |  |  |  |  | 0.127 |
| Negative | 3 (10.7) | 12 (42.9) | 13 (46.4) | 28 (100.0) |  |
| Positive | 2 (10.0) | 12 (60.0) | 6 (30.0) | 20 (100.0) |  |
| Group 2 |  |  |  |  | 0.138 |
| Negative | 3 (15.8) | 10 (52.6) | 6 (31.6) | 19 (100.0) |  |
| Positive | 9 (27.3) | 13 (39.4) | 11 (33.3) | 33 (100.0) |  |
| Group 3 |  |  |  |  | 0.114 |
| Negative | 7 (21.8) | 11 (34.3) | 14 (43.9) | 32 (100.0) |  |
| Positive | 9 (15.5) | 31 (53.4) | 18 (31.1) | 58 (100.0) |  |
| **Progesterone** |  |  |  |  |  |
| Group 1 |  |  |  |  | 0.165 |
| Negative | 3 (10.8) | 13 (46.4) | 12 (42.8) | 28 (100.0) |  |
| Positive | 2 (10.0) | 11 (55.0) | 7 (35.0) | 20 (100.0) |  |
| Group 2 |  |  |  |  | 0.106 |
| Negative | 3 (16.6) | 8 (44.5) | 7 (38.9) | 18 (100.0) |  |
| Positive | 9 (26.5) | 15 (44.1) | 10 (29.4) | 34 (100.0) |  |
| Group 3 |  |  |  |  | 0.113 |
| Negative | 10 (23.3) | 17 (39.5) | 16 (37.2) | 43 (100.0) |  |
| Positive | 6 (12.2) | 28 (57.1) | 15 (30.7) | 49 (100.0) |  |
| **Her-2** |  |  |  |  |  |
| Group 1 |  |  |  |  | 0.191 |
| Negative | 4 (9.6) | 22 (52.4) | 16 (38.0) | 42 (100.0) |  |
| Positive | 0 (0.0) | 1 (25.0) | 3 (75.0) | 4 (100.0) |  |
| Inconclusive | 1 (0.0) | 0 (0.0) | (0.0) | 1 (100.0) |  |
| Group 2 |  |  |  |  | 0.153 |
| Negative | 8 (21.7) | 17 (45.9) | 12 (32.4) | 37 (100.0) |  |
| Positive | 4 (30.7) | 5 (38.6) | 4 (30.7) | 13 (100.0) |  |
| Inconclusive | 0 (0.0) | 0 (0.0) | 0 (0.0) | 0 (0.0) |  |
| Group 3 |  |  |  |  | 0.109 |
| Negative | 11 (19.4) | 27 (47.3) | 19 (33.3) | 57 (100.0) |  |
| Positive | 3 (10.0) | 15 (50.0) | 12 (40.0) | 30 (100.0) |  |
| Inconclusive | 2 (100.0) | 0 (0.0) | 0 (0.0) | 2 (100.0) |  |

*Chi-square/ Fisher’s exact

**Note:** Values in bold indicate statistical significance (p<0.05).

**Table S8:** Correlation between the genotype frequency of polymorphism rs2981582 in gene *FGFR2* and the hormone receptors (per group).

| **Hormone Receptors** | **TT**  **N (%)** | **TC**  **N (%)** | **CC**  **N (%)** | **Total**  **N (%)** | **P value*** |
| --- | --- | --- | --- | --- | --- |
| **Estrogen** |  |  |  |  |  |
| Group 1 |  |  |  |  | 0.114 |
| Negative | 7 (25.0) | 12 (42.9) | 9 (32.1) | 28 (100.0) |  |
| Positive | 7 (35.0) | 8 (40.0) | 5 (25.0) | 20 (100.0) |  |
| Group 2 |  |  |  |  | **0.038** |
| Negative | 0 (0.0) | 14 (73.7) | 5 (26.3) | 19 (100.0) |  |
| Positive | 9 (26.5) | 18 (52.9) | 7 (20.6) | 34 (100.0) |  |
| Group 3 |  |  |  |  | 0.073 |
| Negative | 8 (25.8) | 12 (38.7) | 11 (35.5) | 31 (100.0) |  |
| Positive | 15 (25.0) | 34 (56.6) | 11 (18.3) | 60 (100.0) |  |
| **Progesterone** |  |  |  |  |  |
| Group 1 |  |  |  |  | 0.080 |
| Negative | 6 (21.4) | 13 (46.4) | 9 (32.2) | 28 (100.0) |  |
| Positive | 8 (40.0) | 7 (35.0) | 5 (25.0) | 20 (100.0) |  |
| Group 2 |  |  |  |  | 0.180 |
| Negative | 1 (5.6) | 15 (83.3) | 2 (11.1) | 18 (100.0) |  |
| Positive | 8 (22.9) | 17 (48.5) | 10 (28.6) | 35 (100.0) |  |
| Group 3 |  |  |  |  | **0.025** |
| Negative | 9 (20.9) | 19 (44.2) | 15 (34.9) | 43 (100.0) |  |
| Positive | 14 (28.0) | 28 (56.0) | 8 (16.0) | 50 (100.0) |  |
| **Her-2** |  |  |  |  |  |
| Group 1 |  |  |  |  | 0.082 |
| Negative | 11 (26.2) | 19 (45.2) | 12 (28.6) | 42 (100.0) |  |
| Positive | 2 (50.0) | 1 (25.0) | 1 (25.0) | 4 (100.0) |  |
| Inconclusive | 1 (100.0) | 0 (0.0) | 0 (0.0) | 1 (100.0) |  |
| Group 2 |  |  |  |  | 0.131 |
| Negative | 6 (16.2) | 21 (56.7) | 10 (27.1) | 37 (100.0) |  |
| Positive | 3 (21.4) | 9 (64.3) | 2 (14.3) | 14 (100.0) |  |
| Inconclusive | 0 (0.0) | 0 (0.0) | 0 (0.0) | 0 (0.0) |  |
| Group 3 |  |  |  |  | 0.085 |
| Negative | 16 (27.2) | 25 (42.3) | 18 (30.5) | 59 (100.0) |  |
| Positive | 7 (24.1) | 18 (62.0) | 4 (13.9) | 29 (100.0) |  |
| Inconclusive | 0 (0.0) | 2 (100.0) | 0 (0.0) | 2 (100.0) |  |

*Chi-square/ Fisher’s exact

**Note:** Values in bold indicate statistical significance (p<0.05).

**Table S9:** Correlation between the genotype frequency of polymorphism rs13281615 and the hormone receptors (per group).

| **Hormone Receptors** | **AA**  **N (%)** | **AG**  **N (%)** | **GG**  **N (%)** | **Total**  **N (%)** | **P value*** |
| --- | --- | --- | --- | --- | --- |
| **Estrogen** |  |  |  |  |  |
| Group 1 |  |  |  |  | 0.151 |
| Negative | 7 (25.0) | 15 (53.6) | 6 (21.4) | 28 (100.0) |  |
| Positive | 7 (35.0) | 6 (30.0) | 7 (35.0) | 20 (100.0) |  |
| Group 2 |  |  |  |  | 0.121 |
| Negative | 4 (21.0) | 7 (36.8) | 8 (42.2) | 19 (100.0) |  |
| Positive | 7 (20.6) | 18 (52.9) | 9 (26.5) | 34 (100.0) |  |
| Group 3 |  |  |  |  | 0.100 |
| Negative | 9 (28.1) | 13 (40.6) | 10 (31.3) | 32 (100.0) |  |
| Positive | 16 (26.7) | 32 (53.3) | 12 (20) | 60 (100.0) |  |
| **Progesterone** |  |  |  |  |  |
| Group 1 |  |  |  |  | 0.132 |
| Negative | 7 (25.0) | 16 (57.1) | 5 (17.9) | 28 (100.0) |  |
| Positive | 7 (35.0) | 5 (25.0) | 8 (40.0) | 20 (100.0) |  |
| Group 2 |  |  |  |  | 0.148 |
| Negative | 4 (22.2) | 7 (38.9) | 7 (38.9) | 18 (100.0) |  |
| Positive | 7 (20.0) | 18 (51.4) | 10 (28.6) | 35 (100.0) |  |
| Group 3 |  |  |  |  | 0.102 |
| Negative | 15 (34.1) | 17 (38.6) | 12 (27.3) | 44 (100.0) |  |
| Positive | 11 (22.0) | 28 (56.0) | 11 (22.0) | 50 (100.0) |  |
| **Her-2** |  |  |  |  |  |
| Group 1 |  |  |  |  | 0.169 |
| Negative | 11 (26.2) | 19 (45.2) | 12 (28.6) | 42 (100.0) |  |
| Positive | 2 (50.0) | 1 (25.0) | 1 (25.0) | 4 (100.0) |  |
| Inconclusive | 0 (0.0) | 1 (100.0) | 0 (0.0) | 1 (100.0) |  |
| Group 2 |  |  |  |  | 0.117 |
| Negative | 7 (19.0) | 20 (54.0) | 10 (27.0) | 37 (100.0) |  |
| Positive | 3 (21.4) | 4 (28.6) | 7 (50.0) | 14 (100.0) |  |
| Inconclusive | 0 (0.0) | 0 (0.0) | 0 (0.0) | 0 (0.0) |  |
| Group 3 |  |  |  |  | **0.030** |
| Negative | 14 (23.7) | 29 (49.1) | 16 (27.2) | 59 (100.0) |  |
| Positive | 9 (30.0) | 15 (50.0) | 6 (20.0) | 30 (100.0) |  |
| Inconclusive | 2 (100.0) | 0 (0.0) | 0 (0.0) | 2 (100.0) |  |

*Chi-square/ Fisher’s exact

**Note:** Values in bold indicate statistical significance (p<0.05).

**Table S10:** Correlation between the genotype frequency of polymorphism rs889312 in gene *MAP3K1* and the hormone receptors (per group).

| **Hormone Receptors** | **CC**  **N (%)** | **CA**  **N (%)** | **AA**  **N (%)** | **Total**  **N (%)** | **P value*** |
| --- | --- | --- | --- | --- | --- |
| **Estrogen** |  |  |  |  |  |
| Group 1 |  |  |  |  | 0.141 |
| Negative | 5 (17.8) | 11 (39.2) | 12 (43.0) | 28 (100.0) |  |
| Positive | 3 (15.0) | 7 (35.0) | 10 (50.0) | 20 (100.0) |  |
| Group 2 |  |  |  |  | 0.152 |
| Negative | 3 (15.8) | 6 (31.6) | 10 (52.6) | 19 (100.0) |  |
| Positive | 5 (14.7) | 13 (38.2) | 16 (47.1) | 34 (100.0) |  |
| Group 3 |  |  |  |  | 0.136 |
| Negative | 4 (12.5) | 18 (56.2) | 10 (31.3) | 32 (100.0) |  |
| Positive | 7 (11.7) | 34 (56.7) | 19 (31.6) | 60 (100.0) |  |
| **Progesterone** |  |  |  |  |  |
| Group 1 |  |  |  |  | 0.147 |
| Negative | 4 (14.3) | 11 (39.3) | 13 (46.4) | 28 (100.0) |  |
| Positive | 4 (20.0) | 7 (35.0) | 9 (45.0) | 20 (100.0) |  |
| Group 2 |  |  |  |  | 0.122 |
| Negative | 2 (11.1) | 6 (33.3) | 10 (55.6) | 18 (100.0) |  |
| Positive | 6 (17.1) | 13 (37.1) | 16 (45.8) | 35 (100.0) |  |
| Group 3 |  |  |  |  | 0.124 |
| Negative | 4 (9.1) | 28 (63.6) | 12 (27.3) | 44 (100.0) |  |
| Positive | 7 (14.0) | 25 (50.0) | 18 (36.0) | 50 (100.0) |  |
| **Her-2** |  |  |  |  |  |
| Group 1 |  |  |  |  | 0.173 |
| Negative | 8 (19.0) | 15 (35.7) | 19 (45.3) | 42 (100.0) |  |
| Positive | 0 (0.0) | 3 (75.0) | 1 (25.0) | 4 (100.0) |  |
| Inconclusive | 0 (0.0) | 0 (0.0) | 1 (100.0) | 1 (100.0) |  |
| Group 2 |  |  |  |  | **0.046** |
| Negative | 7 (18.9) | 13 (35.1) | 17 (46.0) | 37 (100.0) |  |
| Positive | 0 (0.0) | 5 (35.7) | 9 (64.3) | 14 (100.0) |  |
| Inconclusive | 0 (0.0) | 0 (0.0) | 0 (0.0) | 0 (0.0) |  |
| Group 3 |  |  |  |  | 0.107 |
| Negative | 6 (10.1) | 33 (55.9) | 20 (34.0) | 59 (100.0) |  |
| Positive | 5 (16.6) | 17 (56.7) | 8 (26.7) | 30 (100.0) |  |
| Inconclusive | 0 (0.0) | 1 (50.0) | 1 (50.0) | 2 (100.0) |  |

*Chi-square/ Fisher’s exact

**Note:** Values in bold indicate statistical significance (p<0.05).

**Table S11:** Correlation between the genotype frequency of polymorphism rs3817198 in gene *LSP1* and the hormone receptors (per group).

| **Hormone Receptors** | **TT**  **N (%)** | **TC**  **N (%)** | **CC**  **N (%)** |  | **P value*** |
| --- | --- | --- | --- | --- | --- |
| **Estrogen** |  |  |  |  |  |
| Group 1 |  |  |  |  | 0.109 |
| Negative | 13 (46.4) | 12 (42.9) | 3 (10.7) | 28 (100.0) |  |
| Positive | 11 (55.0) | 9 (45.0) | 0 (0.0) | 20 (100.0) |  |
| Group 2 |  |  |  |  | 0.193 |
| Negative | 10 (52.6) | 8 (42.1) | 1 (5.3) | 19 (100.0) |  |
| Positive | 18 (53.0) | 15 (44.1) | 1 (2.9) | 34 (100.0) |  |
| Group 3 |  |  |  |  | **0.007** |
| Negative | 19 (61.3) | 11 (35.5) | 1 (3.2) | 31 (100.0) |  |
| Positive | 22 (36.6) | 30 (50.0) | 8 (13.4) | 60 (100.0) |  |
| **Progesterone** |  |  |  |  |  |
| Group 1 |  |  |  |  | 0.186 |
| Negative | 14 (50.0) | 12 (42.8) | 2 (7.2) | 28 (100.0) |  |
| Positive | 10 (50.0) | 9 (45.0) | 1 (5.0) | 20 (100.0) |  |
| Group 2 |  |  |  |  | 0.171 |
| Negative | 10 (55.5) | 8 (44.5) | 0 (0.0) | 18 (100.0) |  |
| Positive | 18 (51.4) | 15 (42.8) | 2 (5.8) | 35 (100.0) |  |
| Group 3 |  |  |  |  | **0.024** |
| Negative | 25 (58.1) | 14 (32.6) | 4 (9.3) | 43 (100.0) |  |
| Positive | 13 (28.2) | 28 (60.9) | 5 (10.9) | 46 (100.0) |  |
| **Her-2** |  |  |  |  |  |
| Group 1 |  |  |  |  | 0.075 |
| Negative | 22 (52.4) | 18 (42.9) | 2 (4.7) | 42 (100.0) |  |
| Positive | 1 (25.0) | 2 (50.0) | 1 (25.0) | 4 (100.0) |  |
| Inconclusive | 0 (0.0) | 1 (100) | 0 (0.0) | 1 (100.0) |  |
| Group 2 |  |  |  |  | 0.201 |
| Negative | 20 (54.0) | 15 (40.6) | 2 (5.4) | 37 (100.0) |  |
| Positive | 6 (42.9) | 8 (57.1) | 0 (0.0) | 14 (100.0) |  |
| Inconclusive | 0 (0.0) | 0 (0.0) | 0 (0.0) | 0 (0.0) |  |
| Group 3 |  |  |  |  | 0.119 |
| Negative | 26 (44.1) | 26 (44.1) | 7 (11.8) | 59 (100.0) |  |
| Positive | 14 (48.3) | 13 (44.8) | 2 (6.9) | 29 (100.0) |  |
| Inconclusive | 0 (0.0) | 2 (100.0) | 0 (0.0) | 2 (100.0) |  |

**Table S12:** Mean age of the polymorphism genotypes.

| **Polymorphism** | **Mean Age** | **Standard Deviation** |
| --- | --- | --- |
| **rs889312** (***MAP3K1*)** |  |  |
| CC | 37.00 | 12.121 |
| CA | 40.28 | 10.830 |
| AA | 36.47 | 10.100 |
| **rs13281615 (8q24)** |  |  |
| AA | 36.69 | 12.120 |
| AG | 38.88 | 10.455 |
| GG | 38.93 | 10.220 |
| **rs2981582 (*FGFR2*)** |  |  |
| TT | 37.20 | 10.552 |
| TC | 38.51 | 10.957 |
| CC | 38.64 | 10.688 |
| **rs3803662 (*TNRC9*)** |  |  |
| TT | 40.15 | 10.592 |
| TC | 38.73 | 11.724 |
| CC | 37.01 | 9.775 |
| **rs3817198 (*LSP1*)** |  |  |
| TT | 37.53 | 11.043 |
| TC | 38.62 | 10.528 |
| CC | 40.31 | 10.435 |
